# Profiles of behavioral, social and psychological well-being in old age and their association with mobility-limitation-free survival

**DOI:** 10.18632/aging.204182

**Published:** 2022-07-18

**Authors:** Marguerita Saadeh, Xiaonan Hu, Serhiy Dekhtyar, Anna-Karin Welmer, Davide L. Vetrano, Weili Xu, Laura Fratiglioni, Amaia Calderón-Larrañaga

**Affiliations:** 1 Aging Research Center, Department of Neurobiology, Care Sciences and Society, Karolinska Institutet and Stockholm University, Solna, Sweden; 2 SWEAH, Department of Health Sciences, Lund University, Lund, Sweden; 3 Functional Area Occupational Therapy and Physiotherapy, Karolinska University Hospital, Stockholm, Sweden; 4 Stockholm Gerontology Research Center, Stockholm, Sweden

**Keywords:** mobility, survival, well-being, successful aging, older adults

## Abstract

Introduction: Successful aging is a multidimensional construct covering behavioral, social, and psychological domains of well-being. We aimed to identify well-being profiles and their association with mobility-limitation-free survival.

Methods: A total of 1488 healthy individuals aged 60+ from the Swedish National study on Aging and Care in Kungsholmen (SNAC-K) were followed-up for 15 years. Mobility limitation was defined as a walking speed <0.8m/s and vital status information was obtained from the National Cause of Death Register. Well-being profiles were derived from different behavioral, social and psychological indicators using latent class analysis among men and women. Cox and Laplace regression models were applied to examine the association with the incidence of a composite endpoint of mobility limitation or death.

Results: At baseline, three well-being profiles (i.e., worst, intermediate, best) were identified, which followed a clear gradient in all behavioral, social and psychological indicators. Compared to those in the worst profile, men and women in the intermediate profile had 27% (HR 0.73; 95% CI 0.56-0.94) and 23% (HR 0.77; 95% CI 0.59-1.00) lower hazard of developing mobility limitation/death. An even greater protective effect was seen among individuals in the best versus worst profile (HR 0.47; 95% CI 0.31-0.70 in men; HR 0.60; 95% CI 0.46-0.78 in women). Men in the intermediate and best profiles survived 1 and 3 years longer without mobility limitation, respectively; these figures were 2 and 3 years for women.

Conclusions: Better profiles of behavioral, social and psychological well-being may prolong mobility-limitation-free survival by at least one year among older adults. Our findings strengthen the evidence-base to achieve successful aging through multi-domain interventions.

## INTRODUCTION

Successful aging is a multidimensional construct covering behavioral, social, and psychological domains of well-being, all amenable to individual actions and public health interventions [1–4]. Even if the multifaceted understanding of older people’s health and well-being is on the rise [5], most studies still focus on single well-being domains. For example, the protective effect of lifestyle factors such as Mediterranean diet [6–8], smoking [9, 10] and leisure activity [11–14] on physical frailty and survival has been extensively explored. Studies have also shown that better social and psychological well-being are associated with a slower decline in physical function [15], lower risk of disability [16], and longer survival [17] among older adults.

Nevertheless, none of these factors alone seems sufficient to explain the great heterogeneity in aging phenotypes, and successful aging may rather result from simultaneously adhering to several of these protective factors [18, 19]. In spite of this, few studies have addressed the complexity and multidimensionality of successful aging by accounting for the coexistence and interactions among several factors and their contribution to old-age health [15, 20–23]. This requires methodological approaches that integrate multiple interrelated indicators to identify those that are shared by groups of older adults with a given aging phenotype [24].

In this study, we used latent class analysis to detect data-driven subgroups of people with similar well-being profiles according to behavioral (diet, smoking, and physical and mental leisure activities), social (social participation, connections, and support) and psychological (life satisfaction, positive and negative affect) well-being indicators, as defined by the Centers for Disease Control and Prevention (CDC) [25]. Given that men and women tend to behave differently on several well-being factors, we performed our analyses stratified by sex. For instance, women are more likely to engage in protective health behaviors (e.g., healthy diet, non-smoking, routine physical checkups), participate in social events and report higher life satisfaction [26, 27], while men tend to be more physically active, feel less lonely and report better psychological well-being, in general [15, 26, 28, 29]. Men and women have also different healthy life expectancies [30].

Most studies looking at indicators of well-being in old age have focused on single outcomes such as frailty, disability or death [31, 32]. Unlike previous studies, we created a mobility-survival aggregate endpoint to convey the importance of not only living longer but also healthier, in consonance with the widespread claim of adding years to life and life to years [33]. Mobility decline precedes disability and premature death, and is therefore considered an optimal early indicator of physical function decay among older adults [34].

The specific aims of this study were: 1) to identify distinct well-being profiles among men and women separately, by using latent class analysis; 2) to determine which of these profiles are associated with the greatest benefit in terms of mobility-limitation-free survival; and 3) to quantify these potential benefits in absolute terms by calculating differences in median age at onset of mobility limitation or death across profiles.

## MATERIALS AND METHODS

### Study population

Data were gathered from the Swedish National Study on Aging and Care in Kungsholmen (SNAC-K; https://www.snac-k.se/), an ongoing population-based study. SNAC-K includes a random sample of adults aged 60 years or older living at home or in institutions in the Kungsholmen district of Stockholm, Sweden, between 2001 and 2004. A random sample of individuals from 11 age cohorts (ages 60, 66, 72, 78, 81, 84, 87, 90, 93, 96 and ≥99 years) were invited to participate in the study. The baseline SNAC-K population included 3363 individuals (73.3% participation rate) who have been followed up regularly: every six years for the young-old cohorts (<78 years) and every three years for the older cohorts (≥78 years). At each study wave, physicians, nurses, and psychologists conducted extensive clinical examinations, interviews, and assessments following standard procedures. SNAC-K data has been linked to the National Patient Register and the Swedish Cause of Death Register to obtain information on medical history and vital status.

This study included data from baseline and four follow-ups of SNAC-K, extending until December 2015 (whereas the death register coverage extended until December 2016). Out of the 3363 participants from SNAC-K baseline, we first excluded 322 individuals (9.6%) with a definite or questionable dementia diagnosis according to the Diagnostic and Statistical Manual of Mental Disorders (4^th^ edition) and 55 individuals (1.8% of the remaining sample) with a Mini-Mental State Examination (MMSE) score <24 because of the limited reliability of their self-reported exposures. We further excluded 704 individuals (23.6% of the remaining sample) with mobility limitation at baseline, i.e., walking speed <0.8 meters per second (m/s), according to an established clinical cut-off [35]. Finally, we excluded 794 individuals (34.8% of the remaining sample) with missing data on any variable (42 individuals with missing data on walking speed, 301 additional individuals with >20% missing data on diet, 9 additional individuals with missing data on smoking, 9 additional individuals with missing data on social connections, 116 additional individuals with missing data on life satisfaction, 285 additional individuals with missing data on positive affect, 32 additional individuals with missing data on negative affect). After applying all exclusion criteria, 1488 functionally healthy participants remained in the study sample (Supplementary Figure 1).

SNAC-K was approved by the Swedish Ethical Review Authority in Stockholm, and written informed consent was obtained from participants or their next of kin.

### Outcome: mobility-limitation-free survival

Walking speed was assessed by trained nurses, whereby participants were asked to walk 6m or 2.4m at a self-selected speed and using a walking aid if needed. The length of the walk was determined by asking participants how fast they normally walked (i.e., normal/fast walkers did the longer walk and slow/very slow walkers did the shorter walk). It was reported as meters per second (m/s), reflecting the speed for whichever length walked [15]. Mobility limitation was defined as having a walking speed below 0.8 m/s, a well-established and clinically validated cut-off [15, 35]. Information on death date was obtained from the National Cause of Death Register hosted and updated annually by the Swedish National Board of Health and Welfare, and linked to SNAC-K data through the personal identification number assigned to Swedish residents.

A composite endpoint, considered to be an indicator of mobility-limitation-free survival, was operationalized by taking into account the time from study entry until the development of mobility limitation (i.e., walking speed <0.8m/s) or death, whichever occurred first.

### Exposures

### Behavioral well-being

### Mediterranean diet

Usual dietary intakes were assessed using a validated self-administered 98-item semi-quantitative food frequency questionnaire (SFFQ). Participants were asked to report their average intake frequency of each food and beverage over the past 12 months on a fixed 9-level scale ranging from never to ≥4 times per day. Zero imputation was applied to replace SFFQ missing items [22]. This is considered a reasonable technique to use with the FFQ, especially for those foods that are not eaten frequently and are thus likely to represent a null consumption [36]. Frequencies were converted into daily consumption and the energy intake was calculated by multiplying these frequencies by a portion size value and by the energy content, based on a food composition database from the National Food Administration using MATs software Version 4.03 (Rudans Lättdata, Västerås, Sweden) [37]. A score indicating the degree of adherence to Mediterranean Diet (MDS: range 0-9) [22] was calculated according to the formula proposed by Trichopoulou et al. [38]. The score was adjusted for total energy intake using the residual method [39]. We further categorized MDS into low, moderate and high adherence according to its tertiles.

### Smoking

Information was obtained from baseline data and smokers and former smokers were asked how long they had smoked and the number of cigarettes smoked per day. Former smokers were also asked at what age they had stopped smoking. We further categorized smoking into current, former and never smokers.

### Physical and mental leisure activities

Participants were asked to specify the type and frequency (i.e., monthly, weekly, less frequently, never) of the leisure activities they regularly engaged in. We grouped the reported activities into physical, mental, and social according to the category they predominantly belonged to, following a procedure described elsewhere [40]. The scores for each type of activity were standardized (i.e., z-scores) based on the baseline mean and standard deviation and were further categorized into low, moderate and high levels according to their tertiles.

Physical leisure activities included: gardening, hiking in the forest/pick berries or mushrooms, hunting/fishing, doing home repairs, and carrying out light exercise (e.g., walking along roads/in parks, walking in the woods, short bicycle rides, light aerobics, golf) or moderate to intense exercise (e.g., jogging, long power walks, heavy duty gardening, long bicycle rides, high intensity aerobics, long distance ice skating, swimming, ball sports other than golf, or other similar activities). Mental leisure activities included: using the internet/playing computer games, painting/drawing/working with clay/pottery, carrying out car or mechanical repairs, and reading books.

### Social well-being

### Social leisure activities (i.e., social participation)

Based on the same procedure described above, the following activities were identified: playing a musical instrument, listening to music, going to sports events, participating in study circles/courses, attending church/revival meetings, participating in volunteer work, going to restaurants/pubs/cafés, dancing, watching TV, playing chess/card games, going to the cinema/theatre/concert, knitting/weaving/sewing, participating in associations/clubs, traveling, reading the newspaper, reading a magazine/journal, going to the bingo and museum/art exhibition. Scores were standardized and further categorized into low, moderate and high according to tertiles.

### Social connections

Participants were asked about their marital status, cohabitation status, parenthood, friendships, and the frequency of direct or remote contacts with parents, children, relatives, neighbors, and friends [15, 41]. Each of these measures were standardized (i.e., z-scores) based on the baseline mean and standard deviation and a global score was obtained by averaging all z-scores. We further categorized social connections into low, moderate and high levels according to tertiles.

### Social support

Participants were asked about the satisfaction concerning their contacts with subjects from their social network, perceived material and psychological support, sense of affinity with potential associations, relatives, and residence area, and feeling part of a group of friends [15, 41]. Each of these measures were standardized (i.e., z-scores) based on the baseline mean and standard deviation and a global score was obtained by averaging all z-scores. We further categorized social support into low, moderate and high levels according to tertiles.

### Psychological well-being

### Life satisfaction

Life satisfaction was assessed through the validated self-reported Life Satisfaction Index A (LSI-A) that captures five different components: zest versus apathy, resolution and fortitude, congruence between desired and achieved goals, positive self-concept, and mood tone. The LSI-A consists of 20 items with an “agree,” “disagree,” or “uncertain” response (range 0-100). A high score indicates that the person takes pleasure from the round of activities that constitutes his or her everyday life, regards life as meaningful and resolutely accepts life as it has been, feels he or she has succeeded in achieving his or her major goals, holds a positive self-image, and maintains happy and optimistic attitudes and mood [15, 41]. We further categorized life satisfaction into low, moderate and high levels according to tertiles.

### Negative affect

Negative affect reflects the extent to which a person feels guilt, anger, or fear, and it considers the following features: distressed, upset, scared, nervous, and afraid. Respondents were asked to report whether and to what extent they had felt in the above-mentioned affective states during the last four weeks. The response options were “not at all,” “a little,” “somewhat,” “quite a bit,” and “very much” (range 5-25) [15, 41]. We further categorized negative affect into low, moderate and high levels according to tertiles.

### Positive affect

Positive affect considers the following affective features: active, inspired, determined, alert, and enthusiastic. Respondents were asked to report whether and to what extent they had felt in the above-mentioned affective states during the last 4 weeks. The response options were “not at all,” “a little,” “somewhat,” “quite a bit,” and “very much” (range 5-25) [15, 41]. We further categorized positive affect into low, moderate and high levels according to tertiles.

### Covariates

Several covariates were considered as possible confounders and measured at baseline: age (continuous), highest level of formal education (elementary school, high school, or university and above), number of chronic diseases (continuous) [42], and Mini-Mental State Examination score (MMSE). In SNAC-K, personality traits (extraversion, neuroticism, and openness to experience) were assessed with a short version of the self-reported NEO Five-Factor Inventory (NEO-FFI) questionnaire [43].

### Statistical analysis

Latent class analysis (LCA) was used to derive the well-being profiles based on the 10 indicators of behavioral, social and psychological well-being assessed at baseline. LCA is a statistical person-centered approach which groups individuals into unobserved classes based on responses to manifest variables. This results in subgroups of individuals similar to each other and distinct from those in other classes [44]. We used the Stata command *gsem lclass* to estimate the latent class models using the maximum-likelihood estimation. Analyses were run separately in men and women. We started with 2 class-solutions, incrementally increasing the number of classes. The ideal number of classes was determined using statistical criteria and interpretations based on theoretical knowledge. Goodness-of-fit indices included Akaike Information Criterion (AIC) and Bayesian Information Criterion (BIC). Participants were assigned to one specific class according to their highest posterior probability. Subsequently, the distribution of all behavioral, social, and psychological factors across the derived classes was examined.

The association between the well-being profiles and the incidence rate of the composite endpoint (i.e., mobility limitation or death) was analyzed through multivariate Cox proportional hazards regression models, from which hazard ratios (HRs) and 95% confidence intervals (CIs) were obtained. The proportional hazard assumption was assessed by regressing the scaled Schoenfeld residuals against survival time. No deviation from the proportional hazard assumption was detected. The models were first adjusted for age and education, and additionally by baseline number of chronic diseases and MMSE score in a second phase. We used Laplace regressions to quantify the differences (in years) in median age at onset of mobility limitation or death (so-called median mobility-limitation-free survival), according to the well-being profiles. In other words, we analyzed the differences in age when 50% of subjects in each well-being profile had developed the composite endpoint. Population-attributable fractions (PAFs) were calculated to estimate the proportion of the composite outcome that would be avoided if all subjects belonged to the best profile, a measure that is valuable from the public health perspective. Because the interpretation of PAFs is more straightforward with binary exposures, all profiles were compared against the one with the greatest mobility-limitation-free survival.

We carried out the following sensitivity analyses. First, in order to verify the robustness of the classes derived from LCA, we performed a data dimension reduction using multiple correspondence analysis (MCA), a generalization of principal component analysis (PCA) for categorical data. We used the obtained dimensions to plot the coordinates for every category of the 10 well-being indicators and their corresponding sum of squared Cosine (Cos^2^). Second, we further plotted the original data on a reduced two-dimensional grid, while labelling the observations with their corresponding well-being profiles obtained from LCA. Finally, we repeated the Cox and Laplace regression analyses: a) additionally adjusting by personality traits, b) excluding participants who were only interviewed at baseline, and c) excluding participants who dropped-out without developing mobility limitation or death.

All analyses were performed using Stata version 15 and R version 3.6.1. with the level of statistical significance set at p <0.05.

## RESULTS

The study population consisted of 1488 individuals with a mean age of 69 years (standard deviation, SD: 8.3) at baseline. The majority were female (59%) and had at least high-school level education (91%) (Table 1). At baseline, the study sample had a median of three chronic diseases (inter-quartile range, IQR: 2;4) and a median MMSE score of 29 (IQR: 29;30). Eligible participants that were excluded due to missingness in any variable (n=794) were older (mean age (SD): 71.8 (8.3)), had a lower education level, more chronic diseases (median: 3; IQR: 2-5) and lower MMSE score (median: 29; IQR: 28-30). Weak-to-moderate correlations were found within and between behavioral, social, and psychological well-being indicators (Table 2). About 47% of the study population developed the composite endpoint during the follow-up (mean: 11 years, SD: 4.0 years) (Table 1). Participants with a walking speed <0.8m/s at baseline, and thus excluded from the sample, were older, had more chronic diseases and reported lower levels of behavioral, social and psychological well-being (Supplementary Table 1).

**Table 1:** Baseline characteristics of the study population by sex.

	**Total population (n=1488)**	**Males (n=609)**	**Females (n=879)**	**p-value**
**Age (mean, SD)**	69.1 (8.3)	68.6 (8.2)	69.5 (8.4)	**0.013**
**Education (%)**				
Elementary	141 (9.5)	58 (9.5)	83 (9.4)	**0.001**
High school	707 (47.5)	256 (42.2)	451 (51.3)	
University	640 (43.0)	295 (48.3)	345 (39.3)	
**Number of chronic diseases, median (IQR)**	3 (2;4)	3 (2;4)	3 (2;4)	**0.031**
**MMSE score, median (IQR)**	29 (29;30)	29 (29;30)	30 (29;30)	0.196
**Mediterranean Diet Score (%)**				
Low	705 (47.4)	271 (44.5)	434 (49.4)	0.178
Moderate	331 (22.2)	144 (23.7)	187 (21.3)	
High	452 (30.4)	194 (31.9)	258 (29.4)	
**Smoking (%)**				
Current	230 (15.5)	86 (14.1)	144 (16.4)	**<0.001**
Former	639 (42.9)	321 (52.7)	318 (36.2)	
Never	619 (41.6)	202 (33.2)	417 (47.4)	
**Physical leisure activity (%)**				
No activity	497 (33.4)	164 (26.9)	333 (37.9)	**<0.001**
Mild activity	510 (34.3)	209 (34.3)	301 (34.2)	
Intense activity	481 (32.3)	236 (38.8)	245 (27.9)	
**Mental leisure activity (%)**				
No activity	545 (36.5)	172 (28.2)	373 (42.4)	**<0.001**
Mild activity	448 (30.1)	182 (29.9)	266 (30.3)	
Intense activity	495 (33.3)	255 (41.9)	240 (27.3)	
**Social leisure activity (%)**				
Low	496 (33.3)	234 (38.4)	262 (29.8)	**0.001**
Moderate	496 (33.3)	200 (32.8)	296 (33.7)	
High	496 (33.3)	175 (28.7)	321 (36.5)	
**Social connections (%)**				
Low	496 (33.3)	175 (28.7)	321 (36.5)	**<0.001**
Moderate	496 (33.3)	199 (32.7)	297 (33.8)	
High	496 (33.3)	235 (38.6)	261 (29.7)	
**Social support (%)**				
Low	496 (33.3)	222 (36.5)	274 (30.9)	0.104
Moderate	496 (33.3)	193 (31.7)	303 (34.5)	
High	496 (33.3)	194 (31.9)	302 (34.4)	
**Life satisfaction (%)**				
Low	550 (37.0)	209 (34.3)	341 (38.8)	0.211
Moderate	572 (38.4)	243 (40.0)	329 (37.4)	
High	366 (24.6)	157 (25.8)	209 (23.8)	
**Negative affect (%)**				
High	482 (32.4)	172 (28.2)	310 (35.3)	**0.012**
Moderate	356 (23.9)	148 (24.3)	208 (23.7)	
Low	650 (43.7)	289 (47.5)	361 (41.1)	
**Positive affect (%)**				
Low	591 (39.7)	243 (39.9)	348 (39.6)	0.676
Moderate	467 (31.4)	197 (32.4)	270 (30.7)	
High	430 (28.9)	169 (27.8)	261 (29.7)	
**Death or mobility limitation during follow-up**	699 (47%)	286 (47%)	413 (47%)	0.993
**Death without mobility limitation during follow-up**	263 (17.7)	133 (21.8)	130 (14.8)	**0.001**
**Mobility limitation (walking speed <0.8 m/s) during follow-up**	436 (29.3)	153 (25.1)	283 (32.2)	**0.003**

SD, standard deviation; IQR, interquartile range; MMSE, Mini Mental State Examination.

p-values for differences observed among males and females estimated using the chi^2^ test.

**Table 2:** Correlations among the different behavioral, social and psychological well-being indicators.

	**MDS**	**Smoking**	**Physical leisure**	**Mental leisure**	**Social leisure**	**Social connection**	**Social support**	**Life satisfaction**	**Negative affect**	**Positive affect**
**MDS**	1.00	-	-	-	-	-	-	-	-	-
**Smoking**	-0.08	1.00	-	-	-	-	-	-	-	-
**Physical leisure**	0.19	0.04	1.00	-	-	-	-	-	-	-
**Mental leisure**	0.17	0.12	0.45	1.00	-	-	-	-	-	-
**Social leisure**	0.15	-0.11	0.31	0.37	1.00	-	-	-	-	-
**Social connection**	0.16	-0.05	0.27	0.31	0.23	1.00	-	-	-	-
**Social support**	0.13	-0.04	0.18	0.25	0.41	0.46	1.00	-	-	-
**Life satisfaction**	0.13	-0.06	0.29	0.34	0.30	0.37	0.41	1.00	-	-
**Negative affect**	-0.04	0.06	-0.03	0.08	-0.03	-0.05	-0.16	-0.24	1.00	-
**Positive affect**	0.14	-0.04	0.32	0.36	0.32	0.25	0.31	0.56	-0.03	1.00

Correlations between and within psychological and social well-being indicators were assessed using the polychoric test.

MDS, Mediterranean Diet Score.

**Figure 1:**
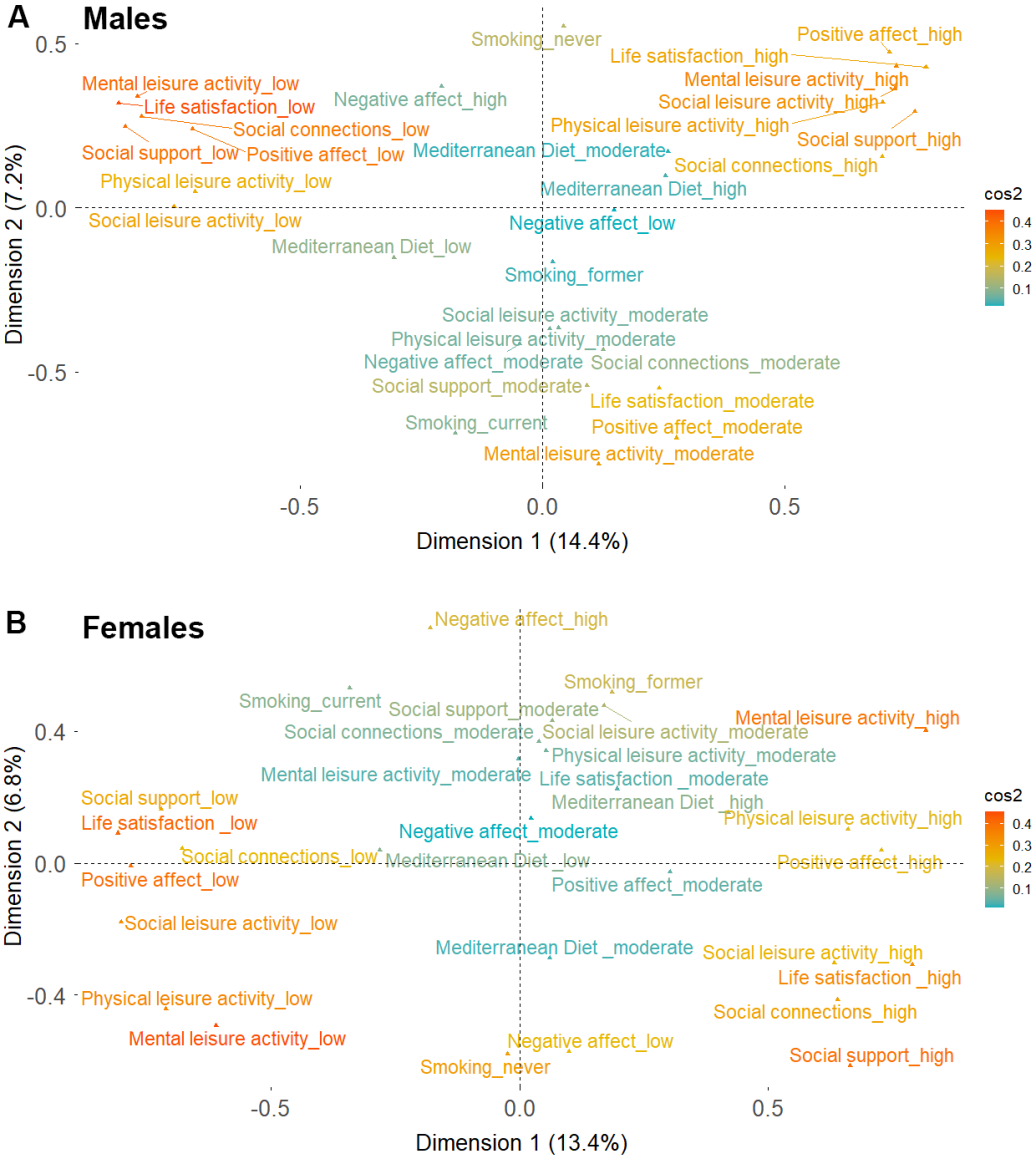
**Two-dimensional coordinates for the different behavioral, social and psychological well-being indicators derived from multiple component analysis.** (**A**) Males (**B**) Females. Cos2 color gradient represents the adequacy of the representation of the different indicators on the two-dimensional map. Cos2 values closer to one indicate a better representation of a variable’s categories over the two-dimensional map.

A three-latent-class solution was identified to be optimal for both men and women, based on the BIC parameter and the interpretation of the identified profiles (Supplementary Table 2). The worst profile (n=151, 25% in men; n=373, 41.4% in women) was characterized by low adherence to MD, a higher proportion of former/never smokers, lowest levels of leisure activity engagement, and lowest levels of social and psychological well-being (Table 3). The intermediate profile (n=313, 51.2% in men; n=237, 28.9% in women) was characterized by low/moderate adherence to MD, a higher proportion of former/never smokers, and moderate levels of social and psychological well-being. Men in this profile had a low/moderate engagement in leisure activities, while women had moderate/high engagement levels. The best profile (n=145, 23.8% in men; n=269, 29.7% in women) was characterized by high adherence to MD, the lowest proportion of current smokers, high engagement with leisure activities, and highest levels of social and psychological well-being (Table 3). Results from the MCA showed that similar levels (i.e., low, moderate, high) of the 10 well-being indicators clustered together confirming the composition of the profiles identified by the LCA (Figure 1 and Supplementary Figure 2).

**Table 3:** Distribution of behavioral, social, and psychological well-being indicators across well-being profiles in males and females.

	**Males (n=609)**			**Females (n=879)**		
	**Worst 25.0%**	**Intermediate 51.2%**	**Best 23.8%**	**Worst 41.4%**	**Intermediate 28.9%**	**Best 29.7%**
**Mediterranean Diet Score (%)**						
Low	61.3	43.9	32.4	59.5	46.4	35.1
Moderate	14.2	22.8	27.1	21.3	21.5	24.4
High	24.5	33.3	40.5	19.2	32.1	40.5
**Smoking (%)**						
Current	15.1	16.8	9.6	19.8	16.9	11.1
Former	50.4	52.3	56.0	28.2	46.7	37.0
Never	34.6	31.9	34.4	52.0	36.3	51.9
**Physical leisure activity (%)**						
No activity	56.0	37.8	0.0	60.4	11.8	17.0
Mild activity	31.0	37.0	27.9	28.9	41.3	31.4
Intense activity	13.0	25.2	72.1	10.7	47.0	51.6
**Mental leisure activity (%)**						
No activity	66.8	32.1	2.4	72.7	17.3	24.8
Mild activity	20.6	46.8	16.4	21.9	35.8	21.5
Intense activity	12.7	21.1	81.3	5.4	46.9	53.8
**Social leisure activity (%)**						
Low	60.2	35.2	1.2	58.3	22.2	9.3
Moderate	30.5	37.9	26.7	25.8	45.2	32.6
High	9.4	26.9	72.2	15.9	32.5	58.1
**Social connections (%)**						
Low	74.8	23.9	10.1	50.6	36.1	6.5
Moderate	20.3	42.4	27.5	29.9	39.9	31.7
High	5.9	33.7	62.4	19.5	24.0	61.8
**Social support (%)**						
Low	78.8	22.9	8.2	50.7	42.5	0.0
Moderate	17.8	44.7	25.2	30.6	43.3	29.3
High	3.4	32.5	66.6	18.6	14.2	70.6
**Life satisfaction (%)**						
Low	87.3	17.8	14.2	67.2	29.3	9.5
Moderate	12.3	55.0	36.5	21.4	42.5	25.4
High	0.4	27.2	49.3	11.5	28.3	66.1
**Negative affect (%)**						
High	41.5	20.0	32.2	27.6	33.0	19.4
Moderate	24.7	23.4	25.8	30.6	38.4	28.5
Low	33.8	56.7	42.0	41.8	28.7	52.2
**Positive affect (%)**						
Low	80.2	30.1	17.5	70.4	25.6	10.2
Moderate	12.8	43.0	30.0	19.4	40.7	36.8
High	7.0	26.3	52.5	10.2	33.8	53.0

**Figure 2:**
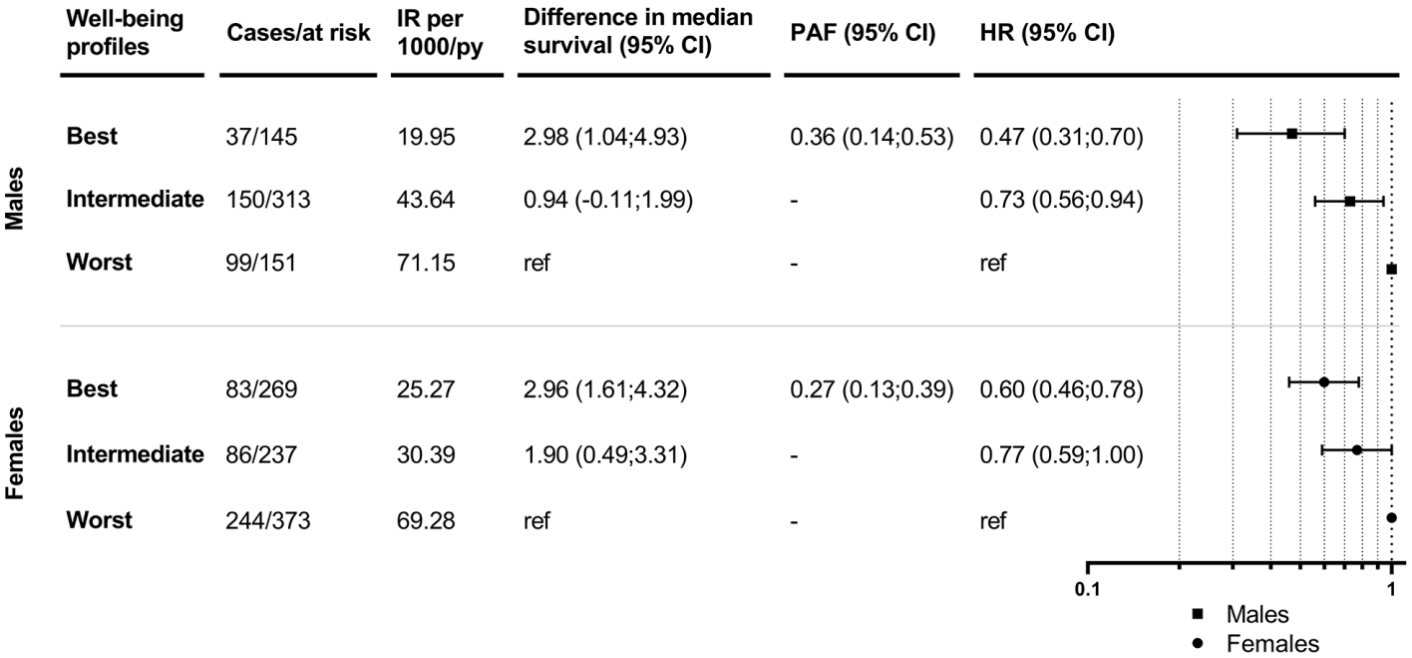
**Associations between well-being profiles and mobility-limitation-free survival in males and females.** IR: incidence rates; PY: person-years; CI: confidence interval; PAF: population attributable fraction; HR: hazard ratio. Models are adjusted for age, education, number of chronic diseases and MMSE score at baseline.

Men and women in the best profile were younger, with higher levels of education, had less chronic diseases at baseline, and had a lower incidence of the composite endpoint compared to those in the worst profile (Supplementary Table 3). The associations between the well-being profiles and the incidence of the composite outcome are shown in Figure 2. After adjusting for potential confounders, men and women in the intermediate profile had a 27% (HR 0.73; 95% CI 0.56-0.94) and a 23% (HR 0.77; 95% CI 0.59-1.00) lower hazard of developing the composite endpoint, respectively, compared to those in the worst profile. An even greater protective effect was seen among individuals in the best profile compared to those in the worst profile (HR 0.47; 95% CI 0.31-0.70 in men; HR 0.60; 95% CI 0.46-0.78 in women). We also show the outcome incidence rates across all well-being indicators and covariates in males and females in Supplementary Table 4.

In agreement with the Cox regressions, results from Laplace regressions showed that men in the intermediate and best profiles survived 1 and 3 years longer without mobility limitations, respectively, compared to those in the worst profile after adjustment for potential confounders (Figure 2). Women in the intermediate and best profiles lived 2 and 3 years longer without mobility limitations, respectively, compared to those in the worst profile. The PAFs for belonging to the intermediate and worst versus the best profile was 36% (95% CI 0.14-0.53) for men and 27% (95% CI 0.13-0.39) for women. Similar trends were observed when further adjusting by personality traits, and when excluding individuals with only baseline information and/or those who dropped-out without developing the outcome (Supplementary Tables 5–7).

## DISCUSSION

In this large community-based cohort study of Swedish older adults aged ≥60 years, we found strong synergistic effects among different behavioral, social, and psychological indicators of well-being, leading to a clear gradient of well-being both in men and women. We also found that individuals in the intermediate and best well-being profiles lived between one to three years longer without mobility limitation compared to those in the worst profile, independent of potential confounders.

The few previous studies looking at multiple indicators of well-being simultaneously have suggested that these tend to be interrelated and to cluster within individuals [21, 24, 45]. For example, a recent study including 15,771 older adults from the Chinese Longitudinal Health Longevity Survey (CLHLS) identified four latent classes in men and women separately based on increasing levels of social engagement, psychological well-being and adherence to positive health behaviors [21]. The WELL study of Australian older adults identified two classes of lifestyle patterns (i.e., healthy and less healthy) based on concomitantly varying levels of healthy diet, physical activity, sedentary behavior, smoking and alcohol consumption [24]. In accordance with these studies, we found that similar levels across 10 different behavioral, social, and psychological well-being indicators clustered within specific well-being profiles, leading to a clear well-being gradient (i.e., worst, intermediate, best) among men and women. Despite the rising evidence supporting a multidimensional construct of successful aging, most longitudinal studies still fail to cover well-being indicators belonging to different domains, as shown by the disproportionate amount of literature focusing exclusively on lifestyle factors [45–48].

Our finding that well-being profiles may considerably prolong mobility-limitation-free survival among older adults has been partially corroborated by previous evidence. Studies carried out with populations from the blue zones constitute a relevant example. These populations are characterized by a high adherence to a healthy diet and physical activity, increased satisfaction with social ties, high social support and psychological well-being, and exceptional longevity without severe disability [49]. A recent study from the seniors-ENRICA cohort concluded that the joint effect of Mediterranean diet, physical activity, rest, social engagement and conviviality conferred the highest protection against frailty [23]. Similarly, in previous studies from SNAC-K, we showed that a higher adherence to Mediterranean diet, especially in combination with recommended levels of physical activity and high levels of social support, can delay the decline in mobility and muscle strength inherent to aging [22] and, that older adults with high levels in both social and psychological well-being have a slower loss of physical function [15]. In another study using SNAC-K, we also found that older adults in the healthiest behavioral profile−concurrently considering lifestyle factors, social network size, and leisure activity participation−lived almost three years longer and developed disability almost four years later compared to those in the least healthy behavioral profile [20]. Compared to the latter study, ours was based on a healthier sample and an outcome (i.e., mobility limitation) that precedes disability. Therefore, our findings further corroborate and complement those from Wu et al. A longitudinal study comprising 10,602 participants aged 40-64 years from the Atherosclerosis Risk in Communities (ARIC) study concluded that those with a healthy lifestyle, defined as following a healthy diet, moderate alcohol and coffee consumption, being physically active, having a normal body weight, and not smoking, had a significantly reduced risk of impaired lower extremity function, and limitations in basic and instrumental activities of daily living than participants with an unhealthy lifestyle [50]. Another longitudinal study of 5,248 American older adults showed that simultaneously adhering to multiple healthy lifestyle factors such as never smoking, moderate alcohol consumption, physical activity, healthy diet, low body mass index, intense social networks, and social support, can compress the number of years lived with disability [51]. Our results advance previous findings by integrating multiple indicators across the behavioral, social and psychological domains of well-being, highlighting their synergistic effects and targeting them as potential modifiable sources of resilience against age-related declines in physical function. Moreover, we created a mobility-survival aggregate endpoint highlighting the importance of not only living longer, but also healthier [33].

Although previous research suggests that non-pharmacological multi-domain interventions can help preserve mental health and cognitive function among older adults [52], there is a clear lack of intervention studies combining behavioral, social and psychological well-being domains in the context of mobility decline prevention in healthy older adults. Instead, most physical function interventions have been based solely on the improvement of nutrition and physical activity and have incorporated well-being and quality of life as primary or secondary outcomes [53–55]. This reflects, among others, the existing asymmetry towards observational studies looking at the reverse association, i.e., impact of physical function on well-being, even if the opposite causal pathway may also be plausible. Along these lines, there is preliminary evidence suggesting that interventions targeting well-being through purposeful activity, i.e., activities that cultivate the pursuit of personal and social goals, feelings of usefulness, and structured community engagement, also impact behavioral, social, and emotional domains [56], but their effect on physical function has not been extensively evaluated. In addition, longitudinal studies exploring the complex mechanisms triggered by the synergies among behavioral, social and psychological well-being factors are warranted, looking beyond their isolated roles in the pathogenesis of functional decline and mortality.

### Strengths and limitations

The strengths of our study include the longitudinal study design, with a relatively large sample size, and with comprehensive information on a number of behavioral, social and psychological well-being indicators. Thanks to the availability of repeated measures of walking speed and vital status throughout the 15-year follow-up, we were able to create a composite endpoint of mobility-limitation-free survival. Moreover, the use of Laplace regressions allowed for the quantification of the years gained in association with belonging to different well-being profiles, enriching the interpretation of our results.

However, several limitations should be considered. Our exposures were assessed only at baseline to avoid reverse causality, which could be challenging among subjects who are less likely to maintain similar levels over time. Additionally, a subsample of the eligible population (35%) lacked data for any study variables, which could lead to selection bias as those with missing data were sicker. Nevertheless, this may be less problematic in our study given that the sample was purposefully selected to be functionally healthy at baseline. Information on all well-being indicators was self-reported, which might have led to misclassification of the exposures. Participants in this study were cognitively healthier and able to self-report their exposure levels, which might have led to an underestimation of the associations in the general population. Finally, our findings, and especially those referring to the well-being profiles, may have a limited transferability beyond the exceptionally healthy and socioeconomically affluent SNAC-K population.

## CONCLUSIONS

While theoretical insights into different models of successful aging are on the rise, empirical evidence from population-based longitudinal data on the complex interplay among the distinct well-being domains and their association with person-centered outcomes, such as mobility-limitation-free survival, is currently lacking. This study addresses such an important gap and provides further evidence to better understand and promote functional independence in community-dwelling older adults through primary prevention multi-domain interventions.

## Supplementary Material

Supplementary Figures

Supplementary Tables
