# Supplementary material for: Profiles of behavioral, social and psychological well-being in old age and their association with mobility-limitation-free survival
Source: Aging (Albany NY). 2022 Jul 18;14(15):5984–6005. doi: 10.18632/aging.204182 (PMC9417239; doi:10.18632/aging.204182)
Supplement: Supplementary Figures [file aging-14-204182-s001.pdf]

SUPPLEMENTARY FIGURES

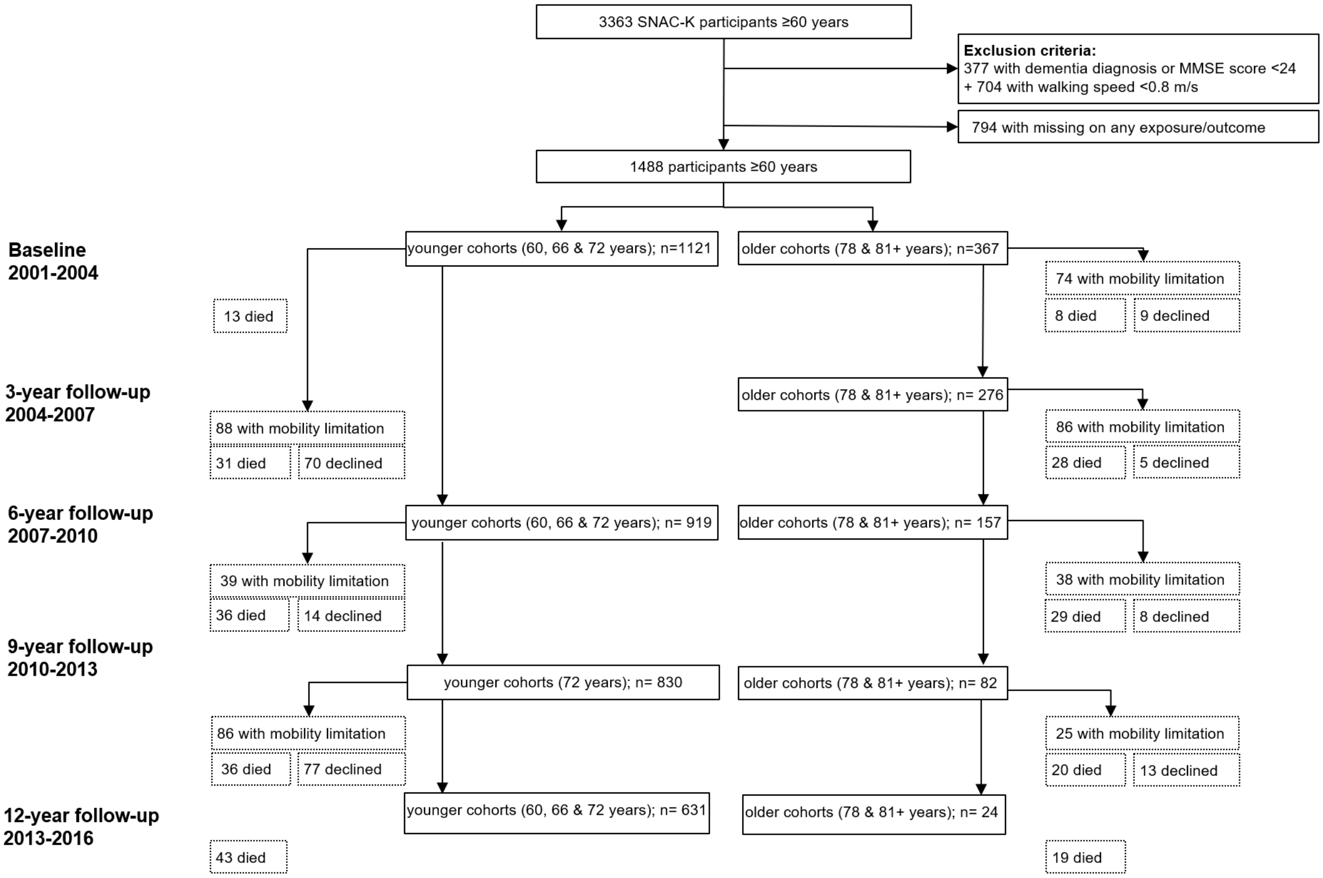

Supplementary Figure 1. Population flow-chart for baseline and follow-up assessments.

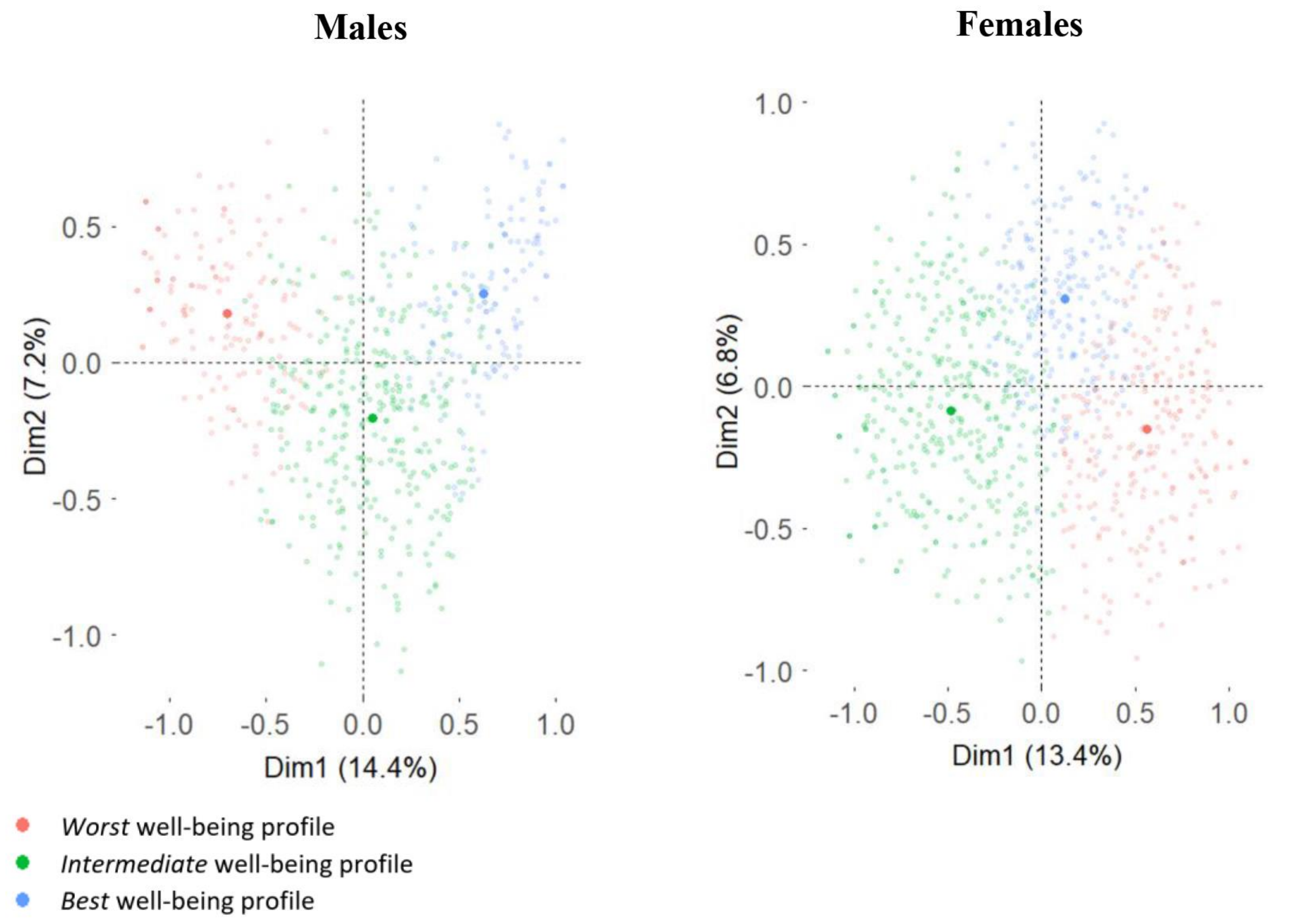

Supplementary Figure 2. Two-dimensional coordinates for all observations in the study sample derived from multiple component analysis. The dot color is assigned in accordance with the well-being profile (derived from LCA) corresponding to that observation. The thickness of the dots is proportional to the number of observations located on a given area of the two-dimensional map.
