# Supplementary material for: Profiles of behavioral, social and psychological well-being in old age and their association with mobility-limitation-free survival
Source: Aging (Albany NY). 2022 Jul 18;14(15):5984–6005. doi: 10.18632/aging.204182 (PMC9417239; doi:10.18632/aging.204182)
Supplement: Supplementary Tables [file aging-14-204182-s002.pdf]

SUPPLEMENTARY TABLES

Supplementary Table 1. Baseline characteristics of the study population by walking speed.

	<0.8 m/s (n=230)	≥0.8 m/s (n=1488)	p-value
**Age (mean, SD)**	**79.3 (8.4)**	**69.1 (8.3)**	**<0.001**
**Sex (%)**			
Male	66 (28.7)	609 (40.9)	**<0.001**
Female	164 (71.3)	879 (59.1)	
**Education (%)**			
Elementary	44 (19.1)	141 (9.5)	
High school	131 (57.0)	707 (47.5)	**<0.001**
University	55 (23.9)	640 (43.0)	
**Number of chronic diseases, median (IQR)**	5 (4;7)	3 (2;4)	**<0.001**
**MMSE score, median (IQR)**	29 (28;29)	29 (29;30)	**<0.001**
**Mediterranean Diet Score (%)**			
Low	144 (62.6)	684 (46.0)	
Moderate	51 (22.2)	335 (22.5)	**<0.001**
High	35 (15.2)	469 (31.5)	
**Smoking (%)**			
Never	127 (55.2)	619 (41.6)	
Former	78 (33.9)	639 (42.9)	**<0.001**
Current	25 (10.9)	230 (15.5)	
**Physical leisure activity (%)**			
No activity	170 (73.9)	403 (27.1)	
Mild activity	42 (18.3)	534 (35.9)	**<0.001**
Intense activity	18 (7.8)	551 (37.0)	
**Mental leisure activity (%)**			
No activity	166 (72.2)	545 (36.6)	
Mild activity	40 (17.4)	395 (26.6)	**<0.001**
Intense activity	24 (10.4)	548 (36.8)	
**Social leisure activity (%)**			
Low	135 (58.7)	438 (29.4)	
Moderate	56 (24.4)	518 (34.8)	**<0.001**
High	39 (17.0)	532 (35.8)	
**Social connections (%)**			
Low	110 (47.8)	463 (31.1)	
Moderate	84 (36.5)	489 (32.9)	**<0.001**
High	36 (15.7)	536 (36.0)	
**Social support (%)**			
Low	116 (50.4)	457 (30.7)	
Moderate	67 (29.1)	506 (34.0)	**<0.001**
High	47 (20.4)	525 (35.3)	
**Life satisfaction (%)**			
Low	137 (59.6)	442 (29.7)	
Moderate	62 (27.0)	538 (36.2)	**<0.001**
High	31 (13.5)	508 (34.1)	
**Negative affect (%)**			
High	86 (37.4)	482 (32.4)	0.175
Moderate	58 (25.2)	356 (23.9)	

Low	86 (37.4)	650 (43.7)	
**Positive affect (%)**			
Low	156 (67.8)	471 (31.7)	
Moderate	50 (21.7)	587 (39.5)	**<0.001**
High	24 (10.4)	430 (28.9)	

SD, standard deviation; IQR, interquartile range; MMSE, Mini Mental State Examination.

Supplementary Table 2. Goodness-of-fit indices of latent class models with 2-4 class solutions.

	Males		Females	
	**AIC**	**BIC**	**AIC**	**BIC**
**2-class solution**	12571.9	12752.8	18303.5	18499.4
**3-class solution**	12473.3	12746.8	18242.5	18538.9
**4-class solution**	12448.82	12815.0	18172.9	18569.6

AIC, Akaike Information Criterion; BIC, Bayesian Information Criterion.
Smaller values indicate improved model fit.

Supplementary Table 3. Baseline characteristics of the study population across well-being profiles in males and females.

	Male (n=609)				Female (n=879)			
	**Worst (n=151)**	**Intermediate (n=313)**	**Best (n=145)**	**p-value**	**Worst (n=373)**	**Intermediate (n=237)**	**Best (n=269)**	**p-value**
**Age (mean, SD)**	71.3 (9.3)	68.8 (7.9)	65.1 (6.0)	0.648	73.0 (8.9)	66.7 (7.1)	67.3 (6.8)	0.457
**Education (%)**								
Elementary	27 (17.9)	27 (8.6)	4 (2.8)		56 (15.0)	13 (5.5)	14 (5.2)	
High school	72 (47.7)	144 (46.0)	40 (27.6)	**<0.001**	215 (57.6)	119 (50.2)	117 (43.5)	**<0.001**
University	52 (34.4)	142 (45.4)	101 (69.7)		102 (27.4)	105 (44.3)	138 (51.3)	
**Number of chronic diseases, median (IQR)**	3 (2;5)	3 (2;4)	2 (1;3)	**0.014**	4 (2;5)	3 (2;4)	3 (2;4)	**<0.001**
**MMSE score, median (IQR)**	29 (29;30)	29 (29;30)	30 (29;30)	**<0.001**	29 (29;30)	30 (29;30)	30 (29;30)	**<0.001**
**Death or mobility limitation during follow-up**	99 (65.6)	150 (47.9)	37 (25.5)	**<0.001**	244 (65.4)	86 (36.3)	83 (30.9)	**<0.001**
**Death without mobility limitation during follow-up**	51 (33.8)	66 (21.1)	16 (11.0)	**<0.001**	79 (21.2)	27 (11.4)	24 (8.9)	**<0.001**
**Mobility limitation during follow-up (walking speed <0.8 m/s)**	48 (31.8)	84 (26.8)	21 (14.5)	**0.002**	165 (44.2)	59 (24.9)	59 (21.9)	**<0.001**

SD, standard deviation; IQR, interquartile range; MMSE, Mini Mental State Examination.
p-values for differences observed among worst, intermediate and best profiles estimated using the chi^2 test.

Supplementary Table 4. Outcome incidence rates by baseline characteristics and well-being indicators in males and females.

	Males (n=609)		Females (n=879)	
	Cases/at risk	IR per 1000/py	Cases/at risk	IR per 1000/py
**Age (years)**				
<78	158/472	27.5	214/649	27.0
≥78	128/137	137.1	199/230	116.2
**Education (%)**				
Elementary	35/58	59.3	57/83	77.0
High school	133/256	49.2	243/451	51.8
University	118/295	34.8	113/345	26.9
**Number of chronic diseases**				
<2	92/282	26.9	88/344	20.2
≥2	194/327	59.4	325/535	61.7
**MMSE score**				
<27	13/16	122.7	14/18	81.2
≥27	273/593	41.5	399/861	42.2
**Mediterranean Diet Score**				
Low	143/277	47.9	232/426	52.5
Moderate	52/132	34.5	89/196	41.0
High	91/200	41.6	92/257	30.2
**Smoking**				
Current	45/86	48.0	68/144	42.3
Former	147/321	41.8	134/318	37.2
Never	94/202	42.2	211/417	47.7
**Physical leisure activity**				
No activity	128/203	66.7	193/294	69.9
Mild activity	80/203	34.4	133/292	40.7
Intense activity	78/203	32.0	87/293	24.1
**Mental leisure activity**				
No activity	140/205	74.4	237/373	66.1
Mild activity	95/201	42.2	99/227	38.2
Intense activity	51/203	20.0	77/279	22.3
**Social leisure activity**				
Low	121/203	59.9	162/293	54.7
Moderate	91/203	40.4	122/294	36.6
High	74/203	30.7	129/292	38.6
**Social connections**				
Low	114/203	55.9	165/293	54.7
Moderate	91/203	40.3	137/293	42.5
High	81/203	33.9	111/293	32.7
**Social support**				
Low	120/203	59.4	157/293	51.0
Moderate	82/203	35.2	127/298	37.8
High	84/203	36.0	129/288	40.3
**Life satisfaction**				
Low	120/209	58.1	202/341	58.9
Moderate	113/243	41.6	118/252	43.4
High	53/157	27.9	93/286	26.7
**Negative affect**				

High	71/172	37.2	112/235	43.5
Moderate	70/148	42.4	115/283	35.3
Low	145/289	46.5	186/361	48.9
**Positive affect**				
Low	142/243	59.7	218/348	64.5
Moderate	89/197	39.3	109/270	35.1
High	55/169	26.9	86/261	27.3

IR, incidence rates; py, person-years; MMSE, Mini Mental State Examination.

Supplementary Table 5. Hazard ratios (HR) and differences in median mobility-limitation-free survival in males and females, and by well-being profiles.

	**Males (n=609)**						**Females (n=879)**					
	**Model I**		**Model II**		**Model III**		**Model I**		**Model II**		**Model III**	
**Results from Cox regressions**												
	**HR (95%CI)**	**p-value**	**HR (95%CI)**	**p- value**	**HR (95%CI)**	**p- value**	**HR (95%CI)**	**p- value**	**HR (95%CI)**	**p- value**	**HR (95%CI)**	**p- value**
**Well-being profiles**												
Worst	**Ref**	-	**Ref**	-	**Ref**	-	**Ref**	-	**Ref**	-	**Ref**	-
Intermediate	0.74 (0.57;0.95)	**0.021**	0.73 (0.56;0.94)	**0.017**	0.75 (0.56;0.99)	**0.048**	0.76 (0.59;0.99)	**0.043**	0.77 (0.59;1.00)	**0.050**	0.95 (0.74;1.22)	0.692
Best	0.46 (0.31;0.69)	**<0.001**	0.47 (0.31;0.70)	**<0.001**	0.48 (0.31;0.74)	**0.001**	0.58 (0.45;0.76)	**<0.001**	0.60 (0.46;0.78)	**<0.001**	0.55 (0.37;0.80)	**0.002**
**Results from Laplace regressions**												
	**β (95%CI)**	**p-value**	**β (95%CI)**	**p-value**	**β (95%CI)**	**p-value**	**β (95%CI)**	**p-value**	**β (95%CI)**	**p-value**	**β (95%CI)**	**p-value**
**Well-being profiles**												
Worst	**Ref**	-	**Ref**	-	**Ref**	-	**Ref**	-	**Ref**	-	**Ref**	-
Intermediate	1.50 (0.13;2.89)	**0.032**	0.94 (-0.11;1.99)	**0.080**	0.93 (-0.04;1.90)	**0.060**	1.95 (0.07;3.82)	**0.042**	1.90 (0.49;3.31)	**0.008**	0.10 (-1.49;1.70)	0.899
Best	3.32 (0.88;5.76)	**0.008**	2.98 (1.04;4.93)	**0.003**	2.99 (1.38;4.60)	**<0.001**	3.06 (1.60;4.52)	**<0.001**	2.96 (1.61;4.32)	**<0.001**	2.63 (0.67;4.59)	**0.008**

HR, Hazard Ratio; CI, Confidence Interval.
Model I: adjusted for age and education.
Model II: additionally adjusted for number of chronic diseases and MMSE score at baseline.
Model III: additionally adjusted for personality traits (openness, extraversion and neuroticism).

Supplementary Table 6. Hazard ratios (HR) and differences in median mobility-limitation-free survival in males and females and by well-being profiles, after excluding subjects interviewed only at baseline.

	Males (n=543)						Females (n=818)					
	**Model I**		**Model II**		**Model III**		**Model I**		**Model II**		**Model III**	
**Results from Cox regressions**												
	**HR (95%CI)**	**p-value**	**HR (95%CI)**	**p- value**	**HR (95%CI)**	**p- value**	**HR (95%CI)**	**p- value**	**HR (95%CI)**	**p- value**	**HR (95%CI)**	**p- value**
**Well-being profiles**												
Worst	**Ref**	-	**Ref**	-	**Ref**	-	**Ref**	-	**Ref**	-	**Ref**	-
Intermediate	0.81 (0.60;1.09)	0.170	0.79 (0.58;1.07)	0.122	0.84 (0.60;0.16)	0.290	0.80 (0.60;1.06)	0.117	0.82 (0.62;1.08)	0.154	0.87 (0.64;1.16)	0.331
Best	0.55 (0.35;0.86)	**0.009**	0.54 (0.35;0.85)	**0.008**	0.58 (1.12;1.17)	**0.027**	0.57 (0.43;0.75)	**<0.001**	0.59 (0.44;0.79)	**<0.001**	0.66 (0.48;0.90)	**0.009**
**Results from Laplace regressions**												
	**β (95%CI)**	**p-value**	**β (95%CI)**	**p-value**	**β (95%CI)**	**p-value**	**β (95%CI)**	**p-value**	**β (95%CI)**	**p-value**	**β (95%CI)**	**p-value**
**Well-being profiles**												
Worst	**Ref**	-	**Ref**	-	**Ref**	-	**Ref**	-	**Ref**	-	**Ref**	-
Intermediate	0.83 (-0.08;1.74)	**0.074**	0.59 (-1.00;2.23)	0.477	0.44 (-1.43;2.32)	0.645	1.74 (0.09;3.38)	**0.038**	1.71 (0.42;3.01)	**0.010**	1.22 (-0.02;2.46)	**0.053**
Best	2.15 (0.06;4.24)	**0.043**	2.04 (-0.47;4.56)	0.112	1.92 (-0.56;4.41)	0.130	2.94 (1.63;4.26)	**<0.001**	2.85 (1.46;4.24)	**<0.001**	2.25 (0.99;3.52)	**<0.001**

HR, Hazard Ratio; CI, Confidence Interval.
Model I: adjusted for age and education.
Model II: additionally adjusted for number of chronic diseases and MMSE score at baseline.
Model III: additionally adjusted for personality traits (openness, extraversion and neuroticism).

Supplementary Table 7. Hazard ratios (HR) and differences in median mobility-limitation-free survival in males and females and by well-being profiles, after excluding subjects who dropped-out without developing the outcome.

	Males (n=549)						Females (n=768)					
	**Model I**		**Model II**		**Model III**		**Model I**		**Model II**		**Model III**	
**Results from Cox regressions**												
	**HR (95%CI)**	**p-value**	**HR (95%CI)**	**p- value**	**HR (95%CI)**	**p- value**	**HR (95%CI)**	**p- value**	**HR (95%CI)**	**p- value**	**HR (95%CI)**	**p- value**
**Well-being profiles**												
Worst	**Ref**	-	**Ref**	-	**Ref**	-	**Ref**	-	**Ref**	-	**Ref**	-
Intermediate	0.76 (0.59;0.98)	**0.036**	0.77 (0.60;0.99)	**0.048**	0.80 (0.59;1.07)	0.125	0.70 (0.54;0.91)	**0.007**	0.69 (0.53;0.90)	**0.006**	0.70 (0.53;0.93)	**0.013**
Best	0.47 (0.31;0.71)	**<0.001**	0.49 (0.33;0.73)	**<0.001**	0.50 (0.32;0.78)	**0.002**	0.48 (0.37;0.63)	**<0.001**	0.49 (0.38;0.64)	**<0.001**	0.51 (0.38;0.68)	**<0.001**
**Results from Laplace regressions**												
	**β (95%CI)**	**p-value**	**β (95%CI)**	**p-value**	**β (95%CI)**	**p-value**	**β (95%CI)**	**p-value**	**β (95%CI)**	**p-value**	**β (95%CI)**	**p-value**
**Well-being profiles**												
Worst	**Ref**	-	**Ref**	-	**Ref**	-	**Ref**	-	**Ref**	-	**Ref**	-
Intermediate	1.38 (-0.02;2.79)	**0.054**	0.80 (-0.16;1.75)	0.103	0.70 (-0.33;1.73)	0.184	2.16 (0.83;3.48)	**0.001**	1.85 (0.37;3.32)	**0.014**	1.35 (0.20;2.51)	**0.022**
Best	3.22 (0.86;5.59)	**0.008**	2.86 (1.15;4.56)	**0.001**	2.78 (1.07;4.48)	**0.001**	3.44 (2.13;4.77)	**<0.001**	3.22 (2.12;4.32)	**<0.001**	2.90 (1.75;4.04)	**<0.001**

HR, Hazard Ratio; CI, Confidence Interval.
Model I: adjusted for age and education.
Model II: additionally adjusted for number of chronic diseases and MMSE score at baseline.
Model III: additionally adjusted for personality traits (openness, extraversion and neuroticism).
